# Novel Combination of Choline with *Withania somnifera* (L.) Dunal, and *Bacopa monnieri* (L.) Wetts Reduced Oxidative Stress in Microglia Cells, Promoting Neuroprotection

**DOI:** 10.3390/ijms241814038

**Published:** 2023-09-13

**Authors:** Vittoria Borgonetti, Nicoletta Galeotti

**Affiliations:** Department of Neuroscience, Psychology, Drug Research, and Child Health (NEUROFARBA), Section of Pharmacology, University of Florence, Viale G. Pieraccini 6, 50139 Florence, Italy; vittoria.borgonetti@unifi.it

**Keywords:** choline, neuroprotection, stress, microglia, adaptogens, oxidative stress

## Abstract

Memory deficit is one of the major negative outcomes of chronic stress. Cholinergic system modulates memory not only through the neuronal cells, but also via interactions with non-neuronal cells, suggesting that microglia can influence synaptic function and plasticity, contributing to cognition and memory function. *Withania somnifera* (L.) Dunal (WS) and *Bacopa monnieri* (L.) Wettst (BM), are traditional herbal medicinal products used for the temporary relief of symptoms of stress. The aim of this study was to investigate whether choline (CLN) activity could be enhanced via an association with adaptogens: WS and BM extracts. First, we optimized an in vitro model of corticotropin-releasing hormone (CRH)-induced oxidative stress on microglial BV2 cells. CRH 100 nM reduced BV2 cell viability and induced morphological changes and neurotoxicity after 24 h of microglia stimulation. Moreover, it induced an increase in the production of reactive oxygen species (ROS) and dysregulated antioxidant protein (i.e., SIRT-1 and NRF-2). The association between choline and adaptogens (CBW) 10 μg/mL counteracted the effect of CRH on BV2 cells and reduced the neurotoxicity produced by BV2 CRH-conditioned medium in the SH-SY5Y cell lines. CBW 200 mg/kg produced an ameliorative effect on recognition memory in the novel object recognition test (NORT) test in mice. In conclusion, combining choline with adaptogen plant extracts might represent a promising intervention in chronic stress associated with memory disturbances through the attenuation of microglia-induced oxidative stress.

## 1. Introduction

While acute stress refers to a short-term and adaptive state, chronic stress is a long-lasting condition related to a dysfunctional and maladaptive response that leads to pathologies. Chronic stress is a major risk factor for developing cardiovascular disorders [1], metabolic alterations [2] and neurological disturbances, such as anxiety, depression, and cognitive impairment [3,4]. The main systems involved in the response to a stressful situation are the hypothalamic–pituitary–adrenal axis, which promotes the release of glucocorticoid hormones from the adrenal gland into the bloodstream, and the autonomic nervous system with an increase in the systemic levels of adrenaline and noradrenaline. These processes affect the immune system, which is activated by alterations in catecholamines and glucocorticoids levels. A persistent stimulation of the immune system activates microglia, resulting in neuroinflammation leading to a dysregulation of normal neuronal activity in the central nervous system (CNS) [5]. Microglia can also be directly activated by noradrenaline and glucocorticoids through the stimulation of ß-adrenergic, mineralocorticoid and glucocorticoid receptors. Indeed, microglia are important players in stress sensing and responses and in the development of associated neurological alterations [6]. Memory deficit is one of major negative outcomes of chronic stress [4]. It is known that cholinergic transmission promotes the mechanisms of mnemonic processes. Muscarinic M1 receptors are highly expressed in the cortex and hippocampus and are prominently involved in memory and cognition [7]. The cholinergic system modulates memory not only via neuronal cells, but also via interactions with non-neuronal cells [8]. This suggests that microglia can influence synaptic function and plasticity, contributing to cognition and memory function [9]. Interestingly, a strong connection was found between chronic neuroinflammation and impaired memory [10]. The complexity of the pathophysiology of chronic stress makes the possible treatment scheme difficult to address. Progress in this field should be a multitarget approach that can simultaneously attenuate microgliosis and potentiate cholinergic transmission. Choline (CLN) is a B-like vitamin nutrient that serves as a precursor for acetylcholine. Lifelong dietary supplementation with CLN has been reported to rescue memory deficits in a mouse model of Alzheimer’s disease [11]. Research on adaptogens has displayed promising results in stress tolerance and improved homeostasis [12]. Adaptogens are defined as nontoxic synthetic or natural medicinal substances that increase the adaptability, resilience, and survival of organisms under stress. Plant adaptogens have multitarget effects on the neuroendocrine–immune system and they can provide pleiotropic activities as a result of the synergistic interactions of constituents, which could not be obtainable via any of their isolated constituents [13]. *Withania somnifera* (L.) Dunal (WS) and *Bacopa monnieri* (L.) Wettst (BM), are among the most investigated medicinal herbs for their adaptogenic activity, together with antioxidant, immunomodulatory, and anti-inflammatory activity [14]. Thus, the aim of this study was to investigate whether CLN activity could be enhanced by an association with adaptogens. Since combining more than one adaptogen showed better efficacy [13], we tested an association of standardized WS and BM extracts in an in vitro model of oxidative stress on microglial BV2 cells produced via CRH stimulation. Then, we tested the activity of the association in the novel object recognition test (NORT) to evaluate the in vivo efficacy.

## 2. Results

### 2.1. CRH 100 nM Reduced BV2 Cell Viability, Induced Morphological Changes after 24 h of Stimulation

**Figure 1:**
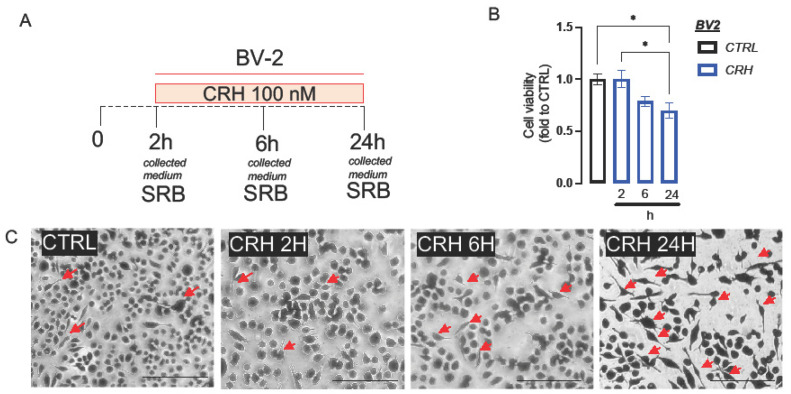
(**A**) Schematical representation of the stimulation of BV2 with CRH 100 nM at 2, 6 and 24 h. (**B**) Cell viability of BV2 cells after CRH at 2, 6 and 24 h, measured by SRB assay (Value normalized on the CTRL group). (**C**) Morphological changes in microgliosis induced by CRH: pro-inflammatory microglia were indicated with red arrow (uncropped figures were reported in Figure S1). Scale bar 100 μM. One-way ANOVA * *p* < 0.05.

The CRH-induced stress model was obtained by stimulating the BV2 microglial cells at different timings: 2 h, 6 h, and 24 h (Figure 1A). The concentration of CRH used is obtained through a previous publication [15,16]. After 24 h, CRH induced a reduction in cell viability in microglia cells, which was not observed after short stimulus (2 h) and only observed with a non-statistically significant tendency after 6 h (Figure 1B CTRL-BV2: 1.0 ± 0.05, CRH 2H: 0.99 ± 0.08, CRH 6H: 0.78 ± 0.05, CRH 24H: 0.68 ± 0.07). No morphological changes were observed after 2 h and 6 h of CRH stimulation (Figure 1C). Differently, after 24 h of CRH stimulation, BV2 assumed a pro-inflammatory phenotype with an increase in cells with small soma and long branches (red arrows; Figure 1C).

### 2.2. CRH Induced Oxidative Stress in BV2

**Figure 2:**
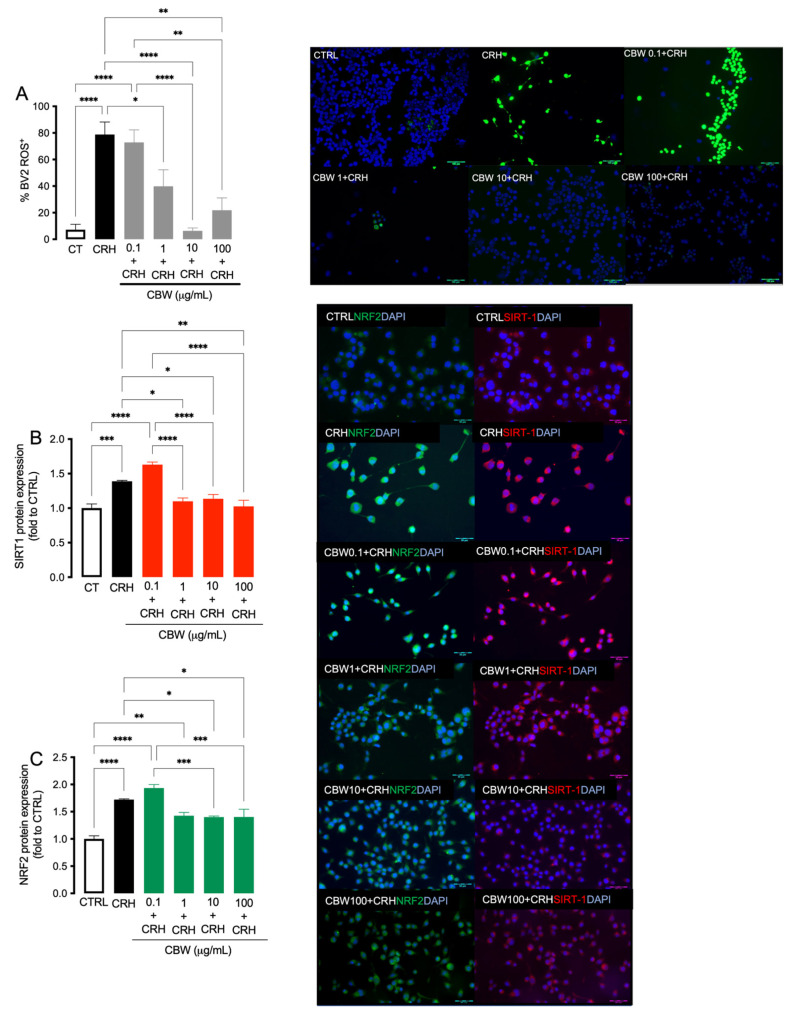
Protective effect of CBW 0.1 1 10 and 100 μg/mL against oxidative imbalance state in CRH-stimulated BV2 cells on BV2 cells. Effect of CBW against ROS production ((**A**), Scale bar 100 μm), SIRT-1 ((**B**), red, Scale bar 50 μm) and NRF2 ((**C**), green Scale bar 50 μm) protein expression. One-way ANOVA **** *p* < 0.0001 *** *p* < 0.001 ** *p* < 0.01 * *p* < 0.05.

**Figure 3:**
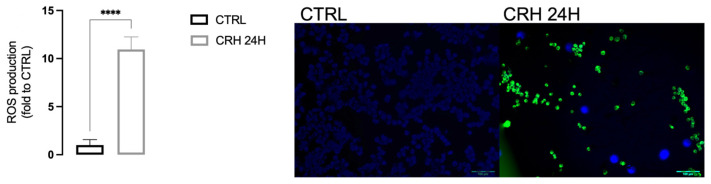
ROS production induced by CRH from BV2 cells and representative images. Representative images have been reported. Scale bar: 100 μM. Student’s *t* test **** *p* < 0.0001.

To investigate whether CRH can reproduce the “oxidative imbalance state” typical of chronic stress, we evaluated the levels of ROS and of the antioxidant enzymes SIRT-1 and NRF-2 (Figure 2) in BV2 cells. CRH induced an increase in ROS production (Figure 3; CTRL-BV2 1.0 ± 0.56, CRH 24H: 10.94 ± 1.31).

### 2.3. Evaluation of the Protective Effect of the Combination CBW in the Oxidative Stress Model on BV2 Cells

**Figure 4:**
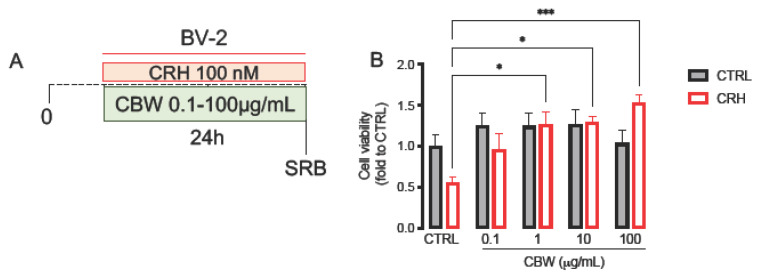
(**A**) Experimental model for testing the efficacy on cell viability of CBW 0.1–100 μM in the CRH-stressed microglia model. (**B**) Evaluation of protective effect of CBW 0.1–100 μM pre-treatment in the oxidative stress model on microglial cells, measured by SRB assay (Value normalized on the basal CTRL group). Gray column = basal; red column = CRH-stimulated. Two-way ANOVA *** *p* < 0.001 * *p* < 0.05.

In the basal condition (without CRH stimulation, Figure 4A), the CBW combination was tested at the concentration of 0.1, 1, 10, and 100 μg/mL on BV2 cells for 24 h, and none of the concentrations induced cytotoxicity (Figure 4B; CTRL BV2: 1.0 ± 0.14, CBW 0.1 BV2: 1.24 ± 0.15; CBW 1 BV2: 1.24 ± 0.16; CBW 10 BV2: 1.26 ± 0.18; CBW 100 BV2: 1.04 ± 0.15).

Then, we tested CBW 0.1–100 μg/mL in the in vitro oxidative stress produced by CRH (Figure 4A). Following stimulation with CRH on BV2 cells, the concentrations of CBW 1 μg/mL, 10 μg/mL, and 100 μg/mL showed significant efficacy in counteracting the toxicity induced by CRH. No significant effects were observed with the concentration of 0.1 μg/mL (Figure 4B, CTRL BV2: 1.0 ± 0.14, CRH: 0.56 ± 0.06; CBW 0.1 + CRH BV2: 0.96 ± 0.19; CBW 1 + CRH BV2: 1.27 ± 0.15; CBW 10 + CRH BV2: 1.29 ± 0.07; CBW 100 + CRH BV2: 1.53 ± 0.09).

### 2.4. Evaluation of the Antioxidant Effect of CBW Association in the CRH-Stimulated BV2

CBW antioxidant effect was evaluated through the investigation of ROS, SIRT-1and NRF2 levels in microglia cells after 24 h of CRH stimulation. CBW 1 μg/mL reduced ROS levels, reaching the peak of activity at 10 μg/mL, which started to disappear at 100 μg/mL (Figure 2A; CTRL BV2: 7.20 ± 4.04, CRH: 78.78 ± 9.43; CBW 0.1 + CRH BV2: 72.85 ± 9.43; CBW 1 + CRH BV2: 39.79 ± 12.43; CBW 10 + CRH BV2: 6.38 ± 2.14; CBW 100 + CRH BV2: 21.85 ± 9.28). Moreover, CRH induced a dysregulation of the antioxidant system, leading to an up-regulation of SIRT1 (Figure 2B) and NRF2 (Figure 2C) compared to the control group. CBW 1, 10 and 100 μg/mL reverted the over-expression produced by CRH, leading to a strong reduction in SIRT-1 (Figure 2B, red: CTRL BV2: 1.0 ± 0.06, CRH: 1.39 ± 0.01; CBW 0.1 + CRH BV2: 1.63 ± 0.03; CBW 1 + CRH BV2: 1.10 ± 0.04; CBW 10 + CRH BV2: 1.13 ± 0.06; CBW 100 + CRH BV2: 1.02 ± 0.08). A similar effect was also observed for NRF2 (Figure 2C, green: CTRL BV2: 1.0 ± 0.05, CRH: 1.72 ± 0.03; CBW 0.1 + CRH BV2: 1.69 ± 0.01; CBW 1 + CRH BV2: 1.42 ± 0.06; CBW 10 + CRH BV2: 1.40 ± 0.01; CBW 100 +CRH BV2: 1.40 ± 0.14). Therefore, the concentration of 10 μg/mL was chosen for the following assays, as it was found to be nontoxic and effective in preventing oxidative stress.

### 2.5. Comparison of the Effect between CBW and Its Principal Constituent (CLN and BW) in Reducing Oxidative Stress Produced by CRH on BV2 Cells

**Figure 5:**
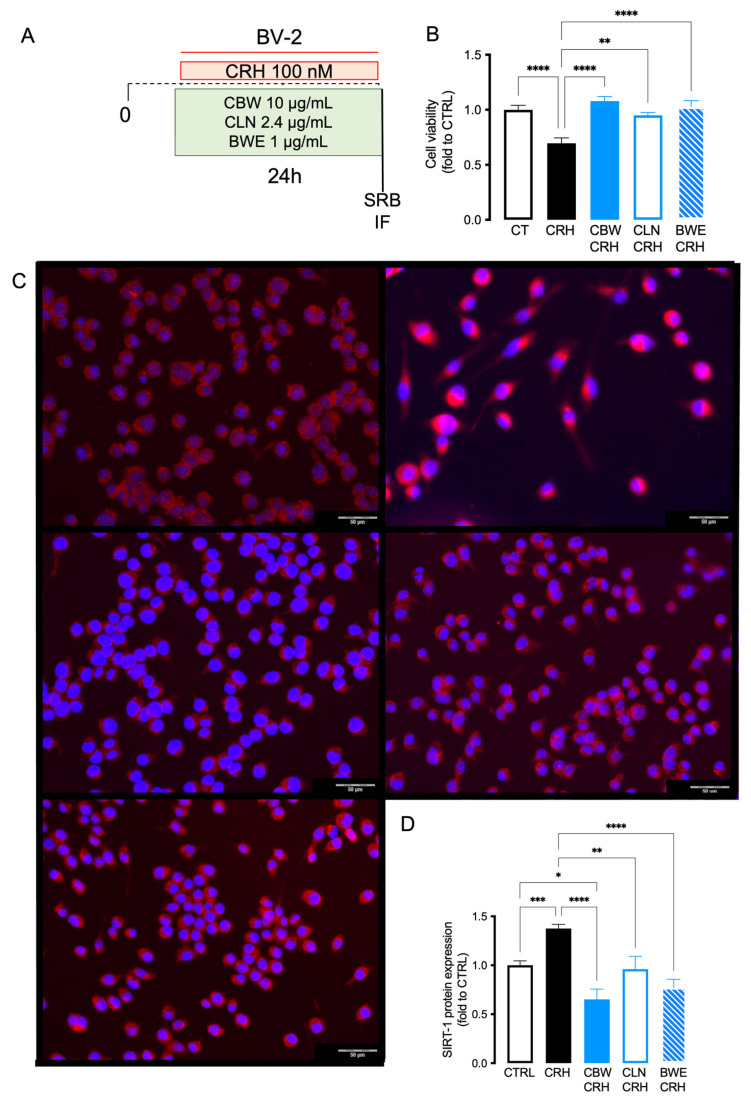
Experimental protocol on BV2 cells (**A**). Comparison of the effect produced by CBW, CLN and BW in CRH-stimulated BV2 on cell viability measured via SRB assay (Value normalized on the basal CTRL group). (**B**) Evaluation of CBW 10 μg/mL efficacy and CLN (4 μg/mL), BW (1 μg/mL) on SIRT-1 protein expression in CRH-stimulated BV2 cells (**C**,**D**). Scale bar: 50 μm. Representative images were reported. One-way ANOVA **** *p* < 0.001 *** *p* < 0.001 ** *p* < 0.01 * *p* < 0.05.

To assess which of the components of CBW is mainly responsible for antioxidant activity in the in vitro model of stress, we performed an immunofluorescence assay to detect the expression of SIRT-1 in microglia cells after CRH stimulation using the concentrations present in the active dose 10 μg/mL of CBW (Figure 5A). In the oxidative stress model, CLN (choline) and BWE (*Withania somnifera* (L.) Dunal (WS) and *Bacopa monnieri* (L.) Wettst (BM) counteracted the cell viability reduction produced by CRH with slight differences in efficacy compared to CBW (Figure 5B; CTRL BV2: 1.0 ± 0.04, CRH: 0.69 ± 0.04; CBW + CRH BV2: 1.079 ± 0.04; CLN + CRH: 0.9488 ± 0.02; BW + CRH: 1.02 ± 0.05). As observed in Figure 5C,D, CBW counteracted the over-expression of SIRT-1 induced by CRH. Similarly, BW reduced the SIRT-1 expression with an efficacy comparable to that of CBW. On the contrary, CLN showed a trend similar to that produced by CRH; indeed, it is only in part able to down-regulate the overexpression of SIRT-1 (CTRL BV2: 1.0 ± 0.04, CRH: 1.37 ± 0.04; CBW + CRH BV2: 0.65 ± 0.10; CLN + CRH: 0.96 ± 0.13; BW + CRH: 0.77 ± 0.08).

### 2.6. Implication of the Cholinergic System in the Neuroprotective Effect of CBW in the In Vitro Model of Stress and Effect on Memory Function In Vivo

At this point, we wanted to investigate whether the protective effect of CBW from oxidative stress on BV2 was also able to induce neuroprotection on SHSY5Y cells. In the baseline condition, CBW (1–100 μg/mL) did not alter SH-SY5Y cell viability (Figure S2).

**Figure 6:**
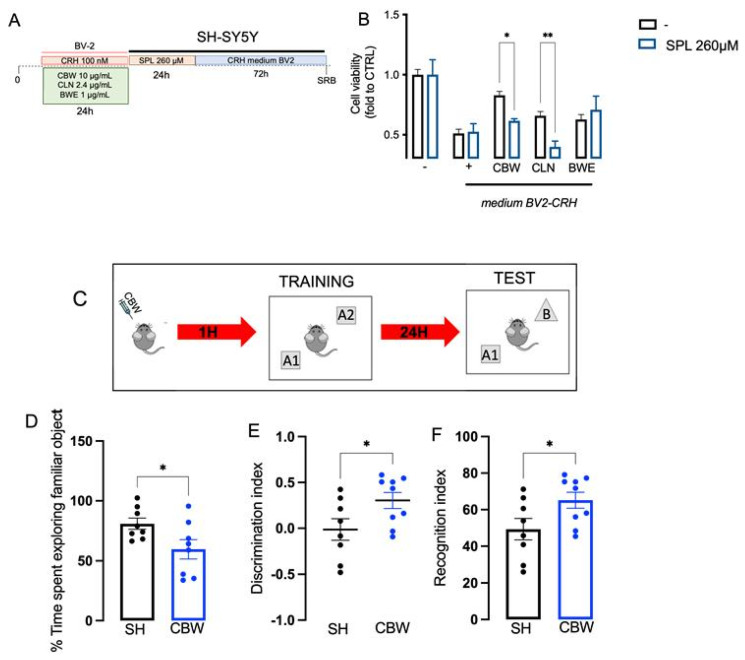
Involvement of cholinergic system in the final effect of CBW (**A**). Scopolamine (SPL) 260 μM did not alter the loss of cell viability induced by CRH, but reverted the effect produced by CBW and CLN. Cell viability was measured using SRB assay (value normalized on the CTRL group without SPL) (**B**), Two-way ANOVA ** *p* < 0.01 * *p* < 0.05. Effect of CBW on novel object recognition test (NORT) after singular oral administration (**C**). During the test session, mice treated with CBW reduced the time spent exploring the familiar object A1 (**D**), and increased the Discrimination index (**E**) and the Recognition index (**F**) compared to the sham group (SH; *n* = 8) and CBW (*n* = 9). Student’s *t* Test * *p* < 0.05.

CBW, CLN and BWE pretreatment on BV2 cells reduced the neurotoxicity in SHSY5Y produced by CRH-conditioned BV2 medium in SH-SY5Y (Figure 6A). To study the involvement of the cholinergic system in the final effect produced by CBW, we pretreated SH-SY5Y with 260 μM scopolamine (SPL), a well-known antimuscarinic drug, for 24 h, followed by the CRH-conditioned BV2 medium for 72 h (Figure 6B). We observed that the pretreatment with SPL did not alter the neurotoxicity produced by the stimulus, but significantly reduced the neuroprotective effect of CBW and CLN. On the contrary, BW did not show any changes in its protective effect after SPL co-treatment (Figure 6B; CTRL-unstimulated BV2: 1.0 ± 0.06, CRH-stimulated BV2: 0.51 ± 0.03, CBW + CRH-stimulated BV2: 0.82 ± 0.03, CLN + CRH-stimulated BV2: 0.65 ± 0.03, BW + CRH-stimulated BV2: 0.67 ± 0.04; SPL-CTRL-unstimulated BV2: 1.0 ± 0.12, SLP-CRH-stimulated BV2: 0.52 ± 0.06, SLP-CBW + CRH-stimulated BV2: 0.61 ± 0.02, SLP-CLN + CRH-stimulated BV2: 0.39 ± 0.04, SLP-BW + CRH-stimulated BV2: 0.70 ± 0.11). As reported in the literature, cholinergic transmission is one of the main factors responsible for memory function.

The NORT was used to investigate the effect of CBW treatment on memory processes (Figure 6C). The initial exploration time recorded during the first test day (Training session9) is reported in the Supplementary Materials (Figure S3).

During the training session, the oral administration of CBW 200 mg/kg reduced the exploration times of the training object (A1) compared to that of control mice (Figure 6D; CTRL: 80.86 ± 4.68, CBW: 59.61 ± 7.99). The discrimination index (Figure 6E; CTRL: −0.013 ± 0.11, CBW: 0.30 ± 0.08) and the recognition index (Figure 6F; CTRL: 49.31 ± 5.88, CBW: 65.17 ± 4.39) of CBW-treated mice were higher than those of control mice, showing that CBW produces an ameliorative effect on recognition memory.

## 3. Discussion

Today, an increasing number of people are experiencing stressful situations and encountering numerous traumatic events that lead to a reduction in their quality of life [17]. Anxiety, depression, cardiovascular disorders, and metabolic alterations are correlated to chronic stress conditions. One of the main debilitating aspects produced by chronic stress is cognitive impairment, which can generate memory loss [18]. Additionally, there are numerous immunological disorders (MS) or neurodegenerative diseases (AD) in which associated memory impairment is observed. Therefore, having a chance to both control the enhancement of cholinergic transmission and to counteract the phenomenon of oxidative stress may be a support to conventional therapies.

CRH is known to be closely related to stress. In fact, its activation leads to the activation of the cascade of pathways involved in the pathophysiological process of stress. Moreover, CRH has recently been investigated as a mediator of the pathways involved in the inflammatory process at the CNS level. As reported, the early stress event provokes microgliosis during a sensitive period of development, with an up-regulation of excitatory synapses on CRH+ neurons in the hypothalamus [19,20]. Indeed, microglia cells express receptors for CRH, leading to an alteration in the biological function [21].

To optimize the in vitro model of oxidative stress induced by CRH on microglia, we used CRH 100 nM, as previously reported [15,16]. This concentration provoked a change in the morphological and functional activity of microglia, particularly increasing the production of IL-18 on BV2 cells [16], which is widely expressed in brain regions involved in emotional regulation, including stress [22]. Indeed, CRH induced a higher release of ROS and an alteration in antioxidant enzyme protein expression. Microglia cells in stress-responsive brain regions up-regulated the expression of several markers associated with their activation [23]. Generally, the oxidative –stress correlation provokes damage to neurons normal transmission, and this is consistent with what we observed in our model, where the oxidative stress produced by CRH in microglia induced damage on neuronal viability and the down-regulation of the expression of SIRT-1/NRF-2, thus highlighting its neurotoxicity. This alteration in normal synaptic transmission can manifest in the patient as anxiety attacks, depression, and, most importantly, memory loss and slowed cognitive processing [24].

One of the most involved systems in the learning and memory process is the cholinergic system. Lifelong choline supplementation significantly reduced amyloid-β plaque load and improved spatial memory trough a reduction in microglial activation [11]. Evidence in the literature indicates that microglial affects memory functions. Resting microglia regulate synaptic pruning, synaptic plasticity, learning and memory, whereas the hyperactivation of microglia, which causes increased the release of proinflammatory cytokines (such as TNF-α, IL-1β, and IL-6), is detrimental [25]. Thus, counteracting proinflammatory microglia and promoting a shift towards the resting microglia could be a way to improve neuroprotection and memory deficits. It has been reported that choline supplementation causes an improvement in mnemonic ability by both restoring normal cholinergic transmission and attenuating microglia activation [11]. Indeed, choline transporter (CTL1) mediated the choline uptake in microglia, promoting the microglia anti-inflammatory polarization [26] and reduced IL-6 production and signaling in LPS-induced neuroinflammation in vivo and in microglia in vitro [27]. 

The role of choline in neuroinflammation is still controversial [28]; thus, for this reason, we investigated the combination of choline (with its effect on the cholinergic mechanism) with BM and WS, two herbal medicines used in traditional medicine as adaptogens to reduce stress with well-known anti-inflammatory and antioxidant activity [13]. Data from a meta-analysis study showed that plant adaptogens could provide significant benefits in the treatment of chronic fatigue, cognitive impairment, and immune protection under stressful conditions [14]. BM showed neuroprotective properties in several in vitro and in vivo models [29]; particularly, it inhibited microgliosis in a mice model of dementia and bacosides [30], and counteracted the cognitive impairment in age-related chronic neuroinflammation in rat [31]. A randomized clinical study performed by Morgan and Stevens observed that the same bacopa extract used in this work at the dose of 300 mg/day for 4 months improved older adults’ memory performance [32].

Similarly, WS has anti-inflammatory and antioxidant activity, as demonstrated both in preclinical and clinical studies [33]. WS extracts inhibited LPS-induced NO and ROS released from BV-2 cells [34] and down-regulated the protein expression of the NF-κB-p65 pathway in primary microglia and LPS-stimulated BV-2, thus reducing microgliosis [35]. Moreover, the administration of a root and leaf extract of WS 400 mg/day for 30 days improved cognitive flexibility, visual memory, and cortisol levels [36], and enhanced attention and short-term/working memory in healthy adults [37]. In our study, these herbal medicine extracts in association with choline reduced the oxidative stress produced by CRH on microglial cells, thus reducing the damage at the SH-SY5Y neuronal cell through a scopolamine-insensitive mechanism, indicating an activity that probably involves the modulation of microgliosis.

## 4. Materials and Methods

### 4.1. Chemicals and Drug Administration

To induce the in vitro model of stress, we used corticotropin-releasing hormone (CRH, Merck, Milan, Italy) 100 nM, as previously performed in dose–response assays [15]. Choline bitartrate (Vitacholine^®,^), *Bacopa monneri* L. aerial part extract (Bacomind™ [38]), containing 22% bacosides, *Withania somnifera* L. bark extract (Ashwapure™, containing 2.5% withanolides, were kindly provided by BiosLine (Ponte San Nicolò, Italy) and were dissolved in serum-free RPMI. The stock solutions were then diluted in complete RPMI.

The fixed combination (CBW) of Choline bitartrate, *Bacopa monneri* L. aerial part extract and *Withania somnifera* L. bark extract was orally administered, using 1% sodium carboxymethyl cellulose (CMC, Sigma-Aldrich, Milan, Italy), an inert excipient to solubilize the ingredients. The CBW was administered p.o. 60 min before the test at the dose of 200 mg/kg. Scopolamine (SPL, Tocris, Milan, Italy), used as muscarinic antagonist, was prepared in serum-free RPMI by making a stock solution and then diluting it in the medium to a concentration of 260 μM.

### 4.2. Cell Culture

BV-2 (murine microglial cells, C57BL/6 Tema Ricerca, Genova, Italy) cells were cultured in 75 cm^2^ flasks (Sarstedt, Verona, Italy) in RPMI, containing 10% heat-inactivated (56 °C, 30 min) fetal bovine serum (FBS, Gibco^®^, Milan, Italy), 1% glutamine, and 1% penicillin-streptomycin solution (Merck), as previously reported [39]. SH-SY5Y (human neuroblastoma cell line) was cultured in a 50/50 mixture of DMEM/F12 Ham’s nutrients medium (Merck), containing 10% heat-inactivated FBS, 1% L-glutamine, and 1% penicillin-streptomycin solution, until confluence (70–80%). Both cell lines were cultured at 37 °C and 5% CO_2_ with a change in culture medium about every other day. EDTA-trypsin solution (Merck) was used for detaching the cells from flasks, and cell counting was performed using a hemocytometer via Trypan blue staining (Merck) [40].

### 4.3. In Vitro Model of Neurotoxicity Induced by Oxidative-Stress-Correlated Microgliosis

BV-2 cells were stimulated with 100 nM CRH, as previously published [15], for 2, 6, and 24 h in RPMI with 3% FBS (Figure 1). Finally, SH-SY5Y neuronal cells have been stimulated with the CRH-conditioned BV2 medium for 72 h (Figure 1). The control group is represented by unstimulated BV2 cells. At the end of the stimulation, biochemical analysis was performed on both cell lines for investigating the oxidative stress produced by the model.

### 4.4. Sulforhodamine B (SRB) Assay

Cell viability and morphological changes were measured using an SRB test, as previously reported [41]. Briefly, the cells were seeded in 96-well plates (2 × 10^4^ cells/well), then fixed with trichloroacetic acid (TCA, Merck). After that, they were treated with SRB (Merck, 0.4% in acetic acid) for 30 min at RT. Finally, the absorbance at 570 nm was recorded using a multiplate reader (Biorad, Milan, Italy). The cell viability was calculated by normalizing the absorbance for each experimental group to the mean of the control group (CTRL). For morphological analysis, the cells were observed with an inverted microscope for cell culture (Leica, Milan, Italy).

### 4.5. Immunofluorescence Staining

Immunofluorescence on BV2 cells was performed as previously reported [39].

Briefly, the BV2 cells were fixed with 4% PFA, and then blocked with BSA 1% in PBS. The primary antibodies against sirtuin-1 (SIRT-1) (Santa Cruz Biotechnology, Dallas, Texas, Cat# sc-74465) and nuclear factor erythroid 2–related factor 2 (NRF-2, Cell signaling, Danver, Massachusettes, mAb #12721) were diluted 1:100 with BSA 5% in PBS-triton 0.03% 2 h at RT. Then, the secondary antibodies labeled with Alexa Fluor^®^ 488 AffiniPure Donkey Anti-Rabbit IgG (H + L) (Jackson ImmunoResearch Labs, Baltimore Pike, West Grove, Pennsylvania, Cat# 711-546-152, RRID: AB_2340619) and Alexa Fluor^®^ 594 AffiniPure Goat Anti-Mouse IgG (H + L) (Jackson ImmunoResearch Labs Cat# 115-585-003) were applied for 1 h. Finally, the slides were mounted with DAPI containing mounting medium (90% glycerol + PBS). Images were acquired using an OLYMPUS BX63F fluorescence microscope connected to a PC with an image acquisition card. The treatments were carried out in three independent experiments (*n* = 3), and the DAPI intensity was calculated by normalizing the values to the mean of the control.

### 4.6. Dosage of ROS Species Level

The production of ROS in BV-2 cells after CRH treatment was quantified using 2′,7′-dichlorodihydrofluorescein diacetate (DCFH-DA, Merck). The cells were initially seeded in 24-well plates (1 × 10^5^ cells/well). Then, the cells were incubated with a 50 μM DCFH-DA probe for 45 min at 37 °C. Fluorescence intensity was measured with the OLYMPUS BX63F fluorescence microscope at excitation 488 nm.

### 4.7. Animals

Male CD1 mice (8 weeks of age, 20 g, Envigo, Varese, Italy) were housed in the vivarium of Ce.S.A.L. (Centro Stabulazione Animali da Laboratorio, University of Florence, Florence, Italy) and used seven days after their arrival. The mice were housed in standard cages, maintained at 23 ± 1 °C with a 12 h light/dark cycle, light on at 7 a.m., and fed with a standard laboratory diet and tap water ad libitum. All tests were conducted during the light phase. The experimental protocol was approved by the ethical committee for animal care and research of the institute University of Florence, under the license of the Italian Ministry of Health (410/2017-PR). The mice were handled in accordance with relevant European Union (Council Directive 2010/63/EU of 22 September 2010 on the protection of animals used for scientific purposes) and international regulations (Guide for the Care and Use of Laboratory Animals, US National Research Council, 2011). All studies involving animals are reported in accordance with the ARRIVE 2.0 guidelines for experiments involving animals. The experimental protocol was designed to minimize the number of animals used and their suffering. G power software was used to perform a power analysis to choose the number of animals per experiment.

### 4.8. Evaluation of Mnemonic Functions with the NORT

Novel object recognition test was used for evaluating memory-related responses in mice, and it was performed as previously reported [42]. The day before the test, the animals were allowed to freely move in an arena, for the acclimation.

During the first session (Day 1-training session), the animals were placed in the center of the arena with two identical objects (A1 and A2) for 5 min. Exploration time assessed as sniffing or touching the object with the nose and mouth was then recorded. Two different operators recorded the time of the animal on each object. To measure long-term memory, the animals were placed back in the same arena 24 h after (day two—retention session) the first test with two objects, the familiar A1 (the same as the day before) and a new object B for 5 min, placed at the same distance as the first day. Objects A1 and B had different shapes, colors, and sizes that had no meaning for the animals. From the test, it was noted for how long the animals observed the novel object, which was calculated with TB/TA1 + TB (Recognition index); the difference in discrimination between object A1 and object B calculated with TB-TA1/TA1 + TB1 (Discrimination index); the percentage of time spent by each animal in exploring the familiar object between the training session (0 h) and the retention session (24 h) calculated as TA1 × 100/5 min (% time spent exploring familiar object). For each experimental group, 8–9 animals per group were used.

### 4.9. Statistical Analysis

For in vitro experiments and in vivo experiments, statistical analysis was performed with one-way or two-way ANOVA, followed by the Tukey or Bonferroni post hoc test. For each test, a value of *p* < 0.05 was considered significant. The data are expressed as the mean ± SEM. The software GraphPad Prism (version 9.0, San Diego, CA, USA) was used in all statistical analyses.

## 5. Conclusions

Chronic stress represents a highly prevalent and disabling pathological condition, leading to the onset of additional associated problems, such as cognitive impairment and memory loss. No drugs are currently able to simultaneously counteract stress and its associated memory deficit. In this work, we proposed an in vitro model of chronic stress-activated microglia capable of generating neurotoxicity through an increase in oxidative stress. In addition, we proposed a new therapeutic scheme to prevent memory loss during chronic stress by combining the activation of the cholinergic transmission with the choline and the attenuation of microglia-induced oxidative stress obtained with the adaptogen herbal medicine BM and WS-standardized extract.

## Data Availability

The data presented in this study are available on request from the corresponding author.

## Supplementary Materials

The following supporting information can be downloaded at: https://www.mdpi.com/article/10.3390/ijms241814038/s1.
