# Supplementary material for: Novel Combination of Choline with Withania somnifera (L.) Dunal, and Bacopa monnieri (L.) Wetts Reduced Oxidative Stress in Microglia Cells, Promoting Neuroprotection
Source: Int J Mol Sci. 2023 Sep 13;24(18):14038. doi: 10.3390/ijms241814038 (PMC10531461; doi:10.3390/ijms241814038)
Supplement: Supplementary file 1 [file ijms-24-14038-s001.zip › ijms-2566907-supplementary.pdf]

Supplementary materials

Novel combination of choline with *Withania somnifera* (L.) Dunal, and *Bacopa monnieri* (L.) Wetts reduced oxidative stress in microglia cells, promoting neuroprotection.

Vittoria Borgonetti [1] and Nicoletta Galeotti [1*]

[1] Department of Neuroscience, Psychology, Drug Research, and Child Health (NEUROFARBA), Section of Pharmacology, University of Florence, Viale G. Pieraccini 6, Florence, 50139, Italy
* Correspondence: Corresponding author: Prof.ssa Nicoletta Galeotti; nicoletta.galeotti@unifi.it

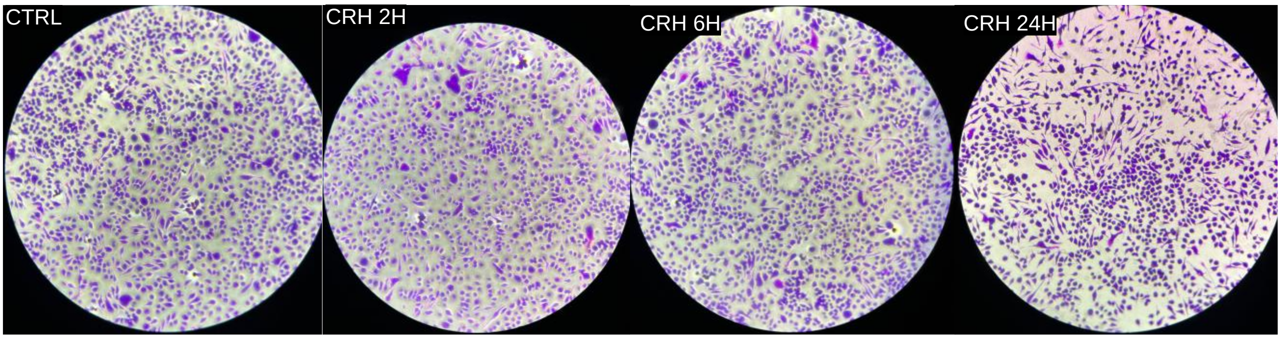

Figure S1: Uncropped images of Figure 1B

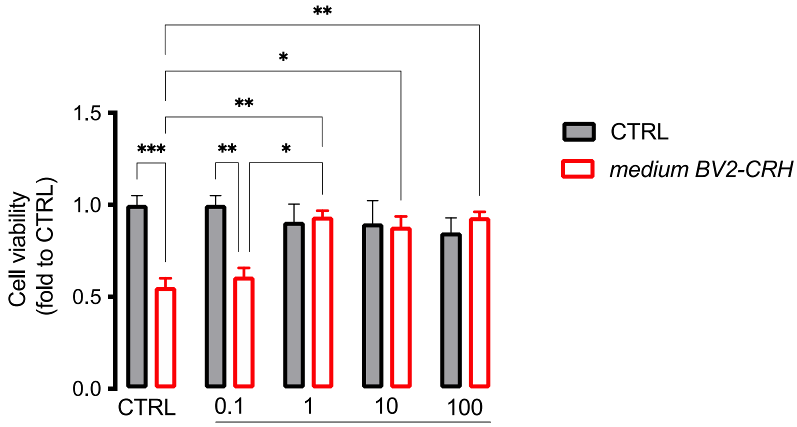

Figure S2 Evaluation of CBW 0.1, 1, 10 and 100 μM pre-treatment in SHSY5Y in basal (gray) and after medium BV2 stimulated with CRH (red). Two-way ANOVA ***p<0.001 **p<0.01 *p<0.05

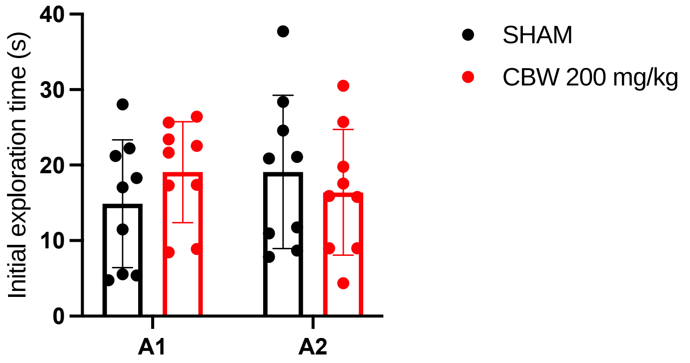

Figure S3 Initial exploration time of SHAM and CBW 200 mg/kg group, recorded during Test Day 1 of NORT test.
